# Nature and characteristics of temperature background effect for interactive respiration process

**DOI:** 10.1038/s41598-017-08871-5

**Published:** 2017-08-17

**Authors:** Guangcai Gong, Xiaorui Deng

**Affiliations:** Department of Building Environment and Energy Engineering, College of Civil Engineering, Hunan University, Changsha, Hunan 410082 China

## Abstract

Indoor air quality (IAQ) is much more crucial to human health than its atmospheric air quality counterpart. Improving indoor air environment requires investigating how different indoor air stability affects airflow trajectory. By presenting both manikin experiment and Computational Fluid Dynamics (CFD) simulation, we find that temperature background effect, i.e., indoor air stability, which is a measure of the nature or attribute of the capacity to keep the original or initial inertia force or inertia transmission state instead of turbulence diffusion or transmission restraining state, i.e., a kind of inertia stability, rather than a turbulence diffusion characteristic stability, is markedly affecting the interactive respiration process. So we define and derive a new parameter called *G*_*c*_ number as a criterion to judge air stability. Furthermore, we find the phenomenon of inertia conjugation. Air stability and inertia conjugation, which named together as temperature background effect, work together on interactive respiration process. This work gives us a re-orientation of temperature difference agents and thus improves human being’s living environment.

## Introduction

**Figure 1:**
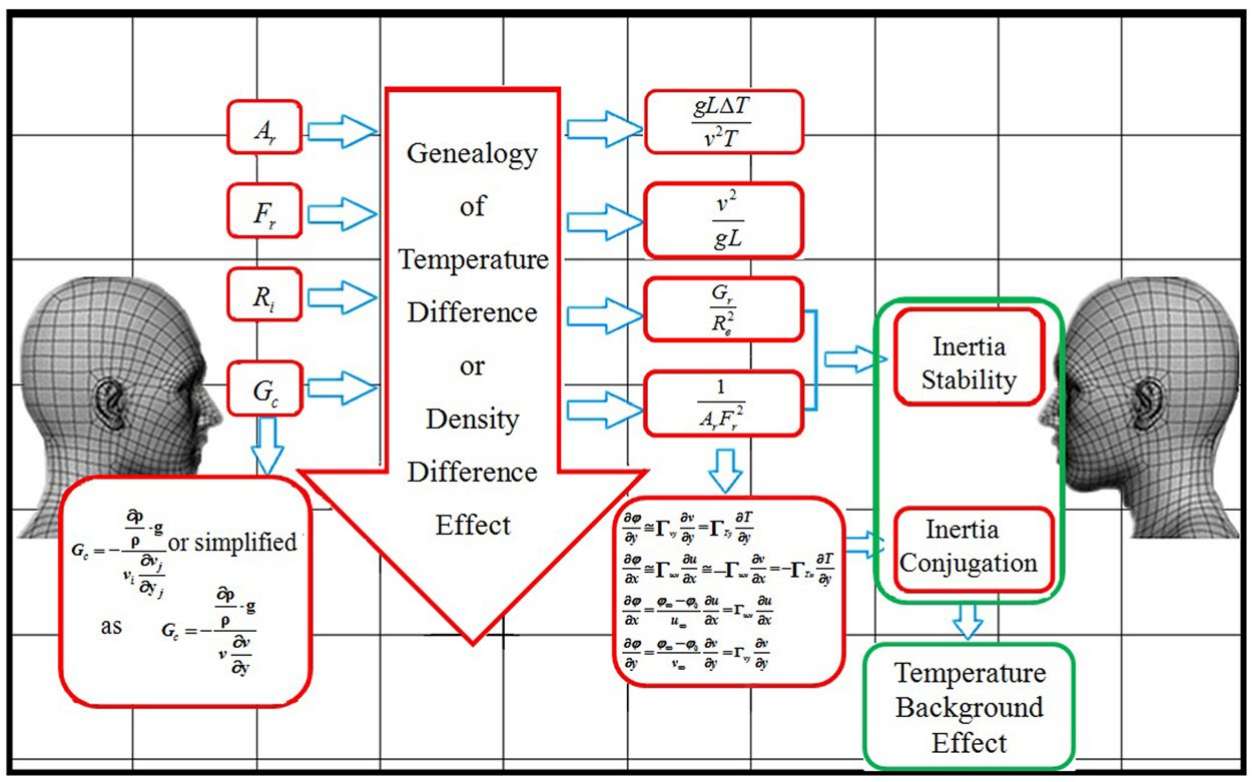
The genealogy of temperature difference or density difference effect. *L* is the height of test room, (m), *v* is the characteristic velocity of airflow, (m/s), *T* is average indoor temperature, (K), *T*_*t*_ is temperature on the top of the room, (K), *T*_*b*_ s temperature at the bottom of the room, (K), Δ*T* is the difference between *T*_*t*_ and *T*_*b*_, (K), *G*_*r*_ is Grashof number, *R*_*e*_ is Reynold number, *ρ* is indoor average density (kg/L), *C* is the smoke concentration value (ppm), *C*_∞_ and *C*_0_ are smoke concentration around the outside research area and wall location (ppm), respectively, Γ_*ux*_ and Γ_*vy*_ are effective or equivalent diffusion coefficient of u along x and y direction, respectively, Γ_*Tx*_ and Γ_*Ty*_ are effective or equivalent diffusion coefficient of *T* along *x* and y direction, respectively, *v*_*i*_ and *v*_*j*_ are component velocity at *i* and *j* direction, respectively, (m/s).

Temperature difference affects atmospheric stability, which attributes to haze accumulation in human habitants. There are some literatures^1–12^ discussing atmospheric stability. However, in limited spaces, such as indoor environment, the same air stability problem still exists, which has a more serious impact on human health, for human beings spend most of their lifetime in interior spaces. Several studies have been made to identify the distribution of pollutants carried in patients’ expiratory airflow in different indoor environment^13–22^, and the effect of different indoor ventilation patterns on the transport characteristics of airborne expiratory droplets has also been studied^23–25^. In general terms, the phenomena of interactive respiration process characteristics under the temperature difference condition are interpreted as diffusion effects so far. Especially, Richardson Number (*R*_*i*_) has been adopted to analyze the relationship between the temperature difference and turbulence diffusion term in atmosphere stability. Nowadays, air stability analysis includes static air stability and *R*_*i*_ analysis that can help us understand the turbulence diffusion character, however, they can not explain the dispersing pattern in main-flow direction, i.e., actually a kind of conjugation phenomena. So, *G*_*c*_ number is put forward in this study as a criterion to judge air stability and we need to rethink the nature of the air stability through interactive respiration process analysis here. We provide a genealogy of the temperature difference or small density difference effects (Fig. 1) and define *G*_*c*_ as the ratio of the density difference (buoyancy) term of Bousinesq’s approximation to the convective inertia force (migrating inertia force) or acceleration term. *G*_*c*_ number is set up by a dimensional analysis and order of magnitude analysis of differential equation based on principles of extended Reynolds Analogy and molecular transport, and is more suitable to reflect the impacts of inertia stability than *R*_*i*_ number in Navier-Stokes or Euler Equations. Here we employ a simplified form of *G*_*c*_, i.e., *G*_*c*_ is the number of the temperature or density difference source to convection or advection term along vertical or gravity direction. In order to make judgment criterion more clear, we also employ ${G}_{c}^{\text{'}}$ as a reciprocal form of *G*_*c*_, which is:

$${G}_{c}^{\text{'}}=\frac{1}{{G}_{c}}={A}_{r}{F}_{r}^{2}$$

where *A*_*r*_ is the Archimedes number, *F*_*r*_ is the Froude number. When *T*_*t*_ < *T*_*b*_, indoor air is in an unstable pattern, *G*_*c*_ = −119.94 < 0, ${G}_{c}^{^{\prime} }=-8.3\times {10}^{-3} < 0$; when *T*_*t*_ ≈ *T*_*b*_, indoor air is in a neutral pattern, *G*_*c*_ = ∞, ${G}_{c}^{^{\prime} }=0$; when *T*_*t*_ > *T*_*b*_, indoor air is in a stable pattern, *G*_*c*_ = 119.94 > 0, ${G}_{c}^{^{\prime} }=8.3\times {10}^{-3} > 0$, and the results are in accordance with the judgments of the dry adiabatic lapse rate. And we also find another phenomenon that there is a kind of conjugation of air pollutant transmission process that along one direction, the pollutant transmission is intense, while along its conjugation direction, the pollutant transmission is weak. As air stability and *G*_*c*_ number mentioned above, we prefer to rethink turbulence is a kind of continuing and compact inertia broken and lost. And the conjugation phenomenon is also a kind of inertia conjugation of transmission process as shown in Fig. 1. So, we name the air stability and conjugation transmission phenomena as the temperature background effect. The temperature background effects would vanish or be 0 when density difference is 0 under any air stability condition.

**Figure 2:**
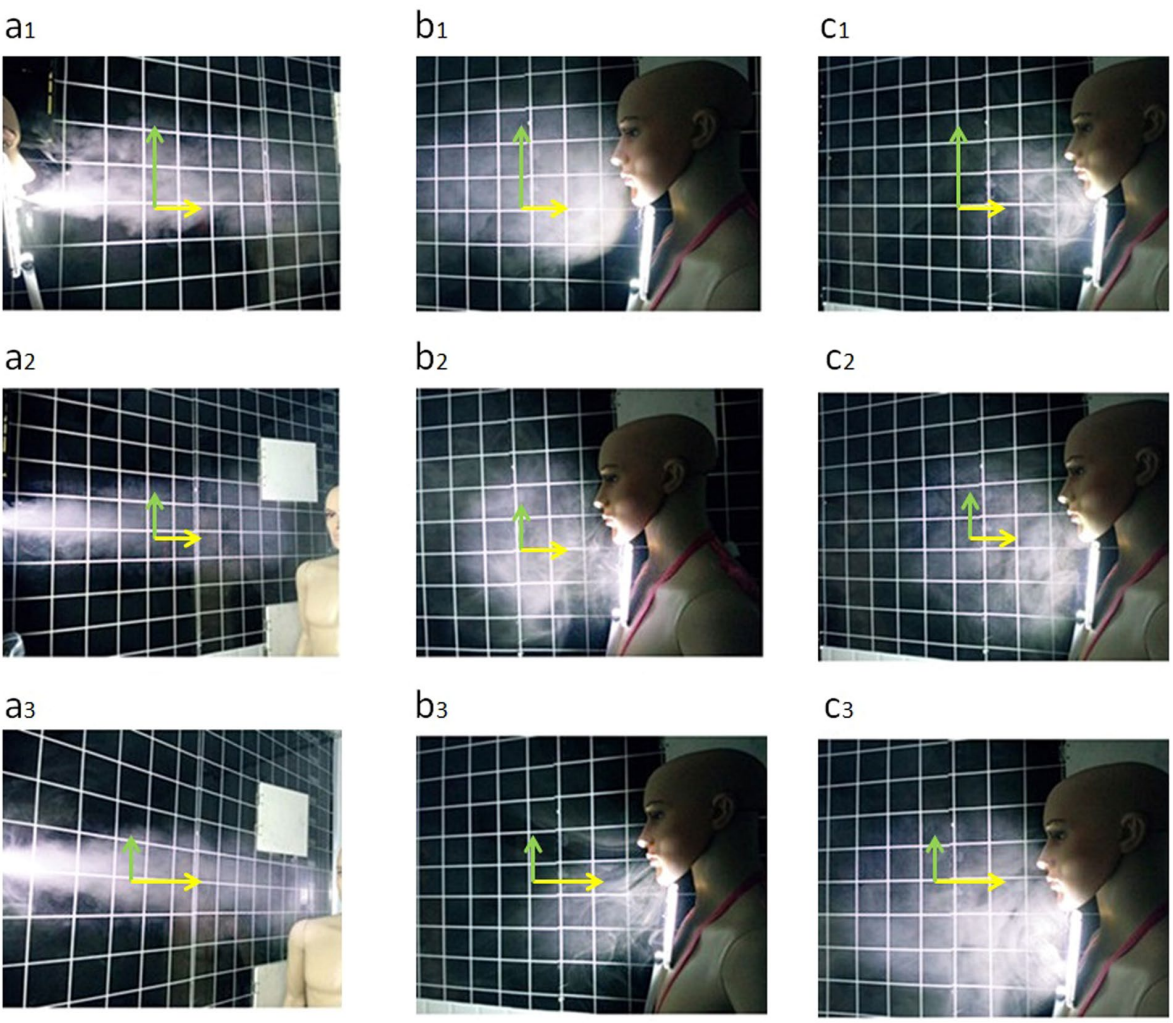
Turbulence diffusion and inertia accumulation caused by temperature background effect under three different inertia stability cases (unstable, neutral, stable) on interactive respiration process. In model experiment, distance between two manikins is 1 m, and the size of mouth and nose is 112 mm^2^ each; the height of the room L is 2.6 m; the initial velocity v_0_ is 3.9 m/s; the temperature around exhaling and inhaling area is 286 K; in unstable case (a_1_, a_2_, a_3_), the vertical temperature gradient is −0.04 T/100 m, with the given temperature on the top of the room T_t_ is 284.1 K, and the given temperature at the bottom of the room T_b_ is 288.1 K, G_c_ = 119.94, ${G}_{c}^{^{\prime} }=8.3\times {10}^{-3}$, which indicates that turbulence diffusion is strengthened, and inertia transmission and accumulation is weakened; in neutral case (b_1_, b_2_, b_3_), the vertical temperature gradient is 0.003 T /100 m, with the top temperature T_t_ is 286.1 K, and the bottom temperature T_b_ is 285.8 K, G_c_ is ∞, ${G}_{c}^{^{\prime} }=0$, which indicates that the turbulence diffusion and inertia transmission and accumulation have not been affected by the temperature or density difference; in stable case (c_1_, c_2_, c_3_), the vertical temperature gradient is 0.04 T/100 m, with the top temperature T_t_ is 288.1 K, and the bottom temperature T_b_ is 284.1 K, G_c_ = −119.94, ${G}_{c}^{^{\prime} }=-8.3\times {10}^{-3}$, which indicates that turbulence diffusion is weakened, and inertia transmission and accumulation is strengthened; (**a**) Temperature background effect on expiration process. The exhaling velocity v_ex_ = 3.9 m/s, inhaling velocity v_in_ = 3.9 m/s, showing turbulence diffusion gets weakened as inertia effect gets stronger. (**b**) Temperature background effect on aspiration process. v_ex_ = 3.9 m/s, v_in_ = 3.9 m/s, showing compared with other cases in this section, smoke in stable type(b_3_), powered by a stronger turbulence diffusion, experiences a more obvious upward movement. (**c**) The effect of aspiration velocity on interactive respiration process. v_ex_ = 3.9 m/s, v_in_ = 1.7 m/s, showing that compared with b, as aspiration velocity decreasing, the amount of smoke inhaled by manikin is also decreasing, leading to inertia accumulation of smoke near the mouth of aspiration manikin.

In order to investigate more details of the interactive respiratory process, we conduct both model experiment and numerical simulation. When exhaling velocity is 3.9 m/s, in exhaling process, the mainstream concentration in unstable case (*G*_*c*_ = −119.94 < 0, ${G}_{c}^{^{\prime} }=-8.3\times {10}^{-3} < 0$) in each position is significantly smaller than that in stable (*G*_*c*_ = 119.94 > 0, ${G}_{c}^{^{\prime} }=8.3\times {10}^{-3} > 0$) and neutral case (*G*_*c*_ *=* ∞, ${G}_{c}^{^{\prime} }=0$). As the mainstream has more intense turbulence diffusion in the vertical direction, the polluted area is larger than that of stable and neutral case where a stronger inertia effect exists. From the perspective of polluted area, in stable case (Fig. 2a_3_), inertia stability restrains the turbulence diffusion, so the polluted area in the vertical direction is the smallest, while in unstable case (Fig. 2a_1_), polluted area is larger because of a weaker inertia stability, of which we can make the most to design a better ventilation system to prevent indoor air pollution. It means that inertia transmission has a kind of directional character under stable air condition. From the perspective of the pollutants intensity, in stable case (Fig. 2a_3_), the smoke concentration maintains a high level, which is more harmful to the polluted area; in unstable case (Fig. 2a_1_), polluted area is larger and the smoke concentration is relatively low, so the harm is smaller.

When inhaling velocity is 3.9 m/s, in inhaling process, the mainstream intensity in unstable case (*G*_*c*_ = −119.94 < 0, ${G}_{c}^{^{\prime} }=-8.3\times {10}^{-3} < 0$) is significantly smaller than that in stable (*G*_*c*_ = 119.94 > 0, ${G}_{c}^{^{\prime} }=8.3\times {10}^{-3} > 0$) and neutral case (*G*_*c*_ *=* ∞, ${G}_{c}^{^{\prime} }=0$). As the smoke experiences a dispersive movement powered by temperature difference distribution which breaks the original or initial inertia state, it spreads around more easily. More stable indoor air can results in inhaling larger amount of pollutants with bigger inhaling velocity (Fig. 2b_3_). From the perspective of polluted area and pollutants intensity, experiment outcomes turn out to be the same with that of exhaling process.

When inhaling velocity is 1.7 m/s, in inhaling process, the characteristics of air stabilities of smoke transmission are the same with that under condition of 3.9 m/s inhaling velocity; but more smoke accumulates together in front of manikin face under the inhaling velocity is 1.7 m/s (Fig. 2b,c). It shows that holding breath can be an effective way to adjust smoke intensity inhaled. In unstable case (Fig. 2c_1_), smoke has the same upward movement trend instead of staying in inhalation area, leading to the low smoke intensity near manikin’s nose; in stable case (Fig. 2c_3_), smoke stays in inhalation area near the manikin’s nose with less dispersion because of the inertia stability, causing the highest smoke intensity. The result showed in neutral type (Fig. 2c_2_) lies between unstable and stable case.

Therefore, we can conclude that air stability or inertia stability plays a decisive role on indoor pollutants dispersion. Two facts are found in Fig. 2. First, airflow of the same exhaling velocity gets a further distance along mainstream direction in stable condition(Fig. 2a_1_,a_2_,a_3_). This is truly inertia transmission phenomena, which means the air stability is a kind of capacity of keeping the original or initial state of inertia force. Second, there is a kind of inertia accumulation effect when exhaling air flow reaches manikin because of the inertia stagnation and rebound (Fig. 2b), and the inertia accumulation effect is more obvious while indoor air maintains a more stable state (Fig. 2c). Furthermore, bigger inhaling velocity can make manikin gets more smoke or pollutants when air stability is stronger (Fig. 2b_1_,b_2_,b_3_). We think that, first of all, this is a kind of temperature difference or small density difference effect to convective or migrating inertia force. Air stability is a kind of map of temperature difference agent or density difference agent which affects the respiration airflow along two directions, and keeps the original or initial inertia force or inertia transmission state other than turbulence diffusion or transmission state. Secondly, there is a kind of conjugation phenomena that there is a pair or couple of conjugation transmission of pollutant when along the mainstream direction, convective or migrating transmission is stronger, i.e., the inertia transmission, and along the conjugation direction, the inertia transmission or turbulence diffusion is weak (the pairs of the arrows with yellow and green color in Fig. 2). That is also this kind of temperature difference or small density difference effect, i.e., the temperature background effect.

Indoor air stability is classified into three different types: unstable, neutral and stable type. Four CFD cases are conducted in this study to represent different indoor air stability types, with one case as a control experiment whose g = 0 m/s^2^.

**Figure 3:**
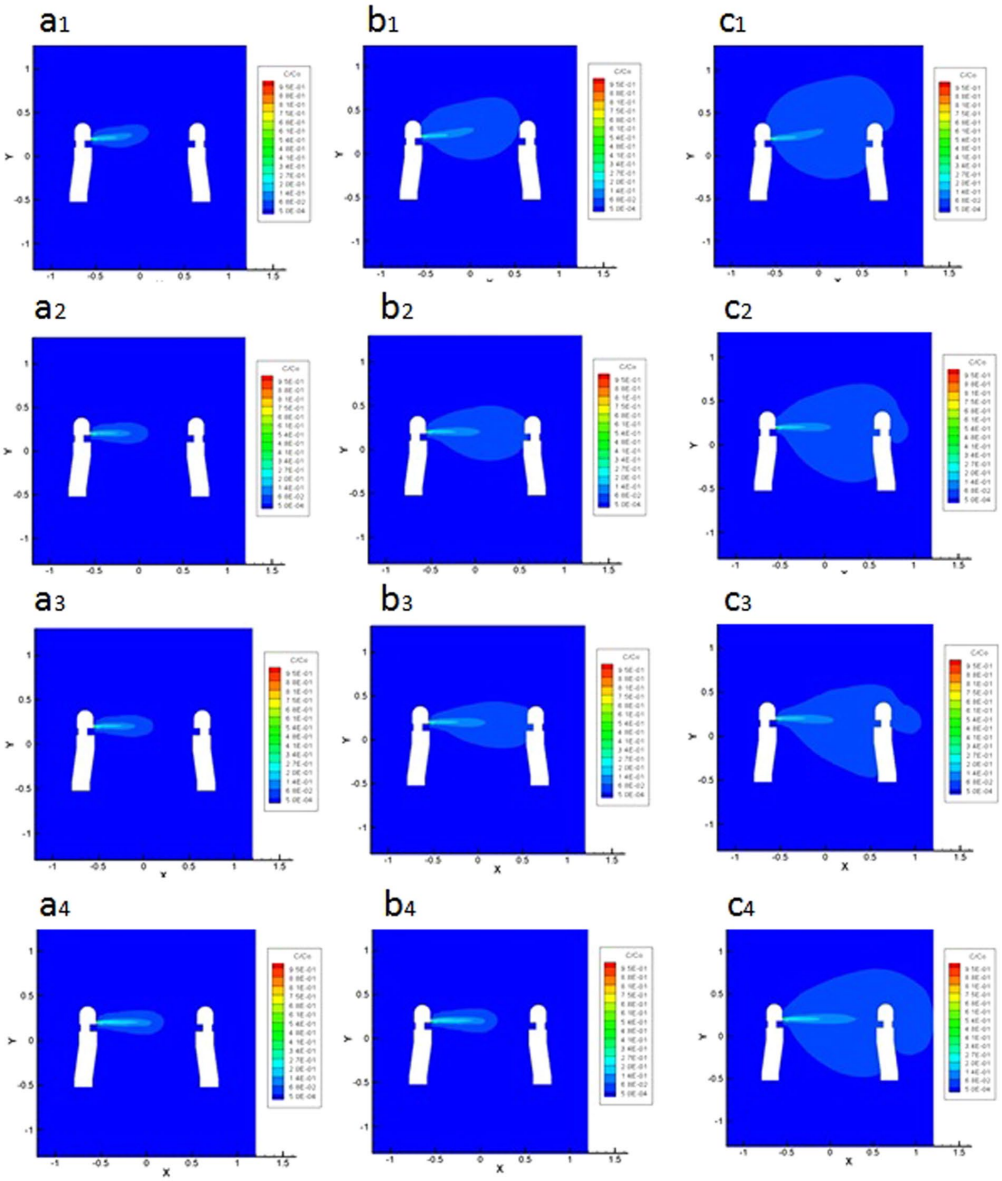
The cloud image of mass concentration distribution of CO_2_ under unstable case (case 1), neutral case (case 2), stable case (case 3) and 0 gravity case (case 4), formed on the section of z = −0.45 m, at t = 1 s, t = 4 s, t = 10 s; a geometric model in 1:1 proportion with actual laboratory is established to ensure consistency with the experimental results. RNG *k* − *ε* viscosity model and Boussinesq approximation is adopted; four cases are related to two gravity condition: one has the acceleration of gravity of −9.8 m/s^2^ (case 1, case 2, case 3),and the other one does not consider the effect of gravity (case 4, relating to unstable of T_t_ < T_b_, neutral of T_t_ = T_b_, stable condition of T_t_ > T_b_); SIMPLEC method and the second order upwind difference scheme are adopted in calculating process; standard wall function is adopted; residuals of k and *ε* are both 10^−3^, and the rest variables are both 10^−6^. (**a**_**1**_,**a**_**2**_,**a**_**3**_) When t = 1 s, cloud images of three cases (case 1,case 2,case 3); (**b**_**1**_,**b**_**2**_,**b**_**3**_) when t = 4 s, cloud images of three cases (case 1,case 2,case 3); (**c**_**1**_,**c**_**2**_,**c**_**3**_) when t = 10 s, cloud images of three cases (case 1,case 2,case 3); (**a**_**4**_,**b**_**4**_,**c**_**4**_) cloud images of 0 gravity case, where G_c_ number is 0.

We can find in Fig. 3 that the CFD simulation shows high consistency with experiment analysis above mentioned in Fig. 2. In unstable case (Fig. 3a_1_,a_2_,a_3_), *G*_*c*_ = −119.94 < 0, ${G}_{c}^{^{\prime} }=-8.3\times {10}^{-3} < 0$, the turbulence diffusion is promoted, and smoke has a slower speed of convective or migrating inertia transmission and a smaller concentration; in stable case (Fig. 3b_1_,b_2_,b_3_), *G*_*c*_ = 119.94 > 0, ${G}_{c}^{^{\prime} }=8.3\times {10}^{-3} > 0$, the convective or migrating inertia transmission is promoted, so smoke in the X axial direction has a farther moving distance and a higher concentration; when G_c_ number is 0 (Fig. 3a_4_,b_4_,c_4_), the characteristics of inertia force and turbulence diffusion are not affected by different indoor air distribution, and the transmission in 0 gravity case is always faster than that in neutral case when gravity exists, but the spread trajectory is basically the same, showing that if there is no gravity, there is no inertia transmission process variation.

**Figure 4:**
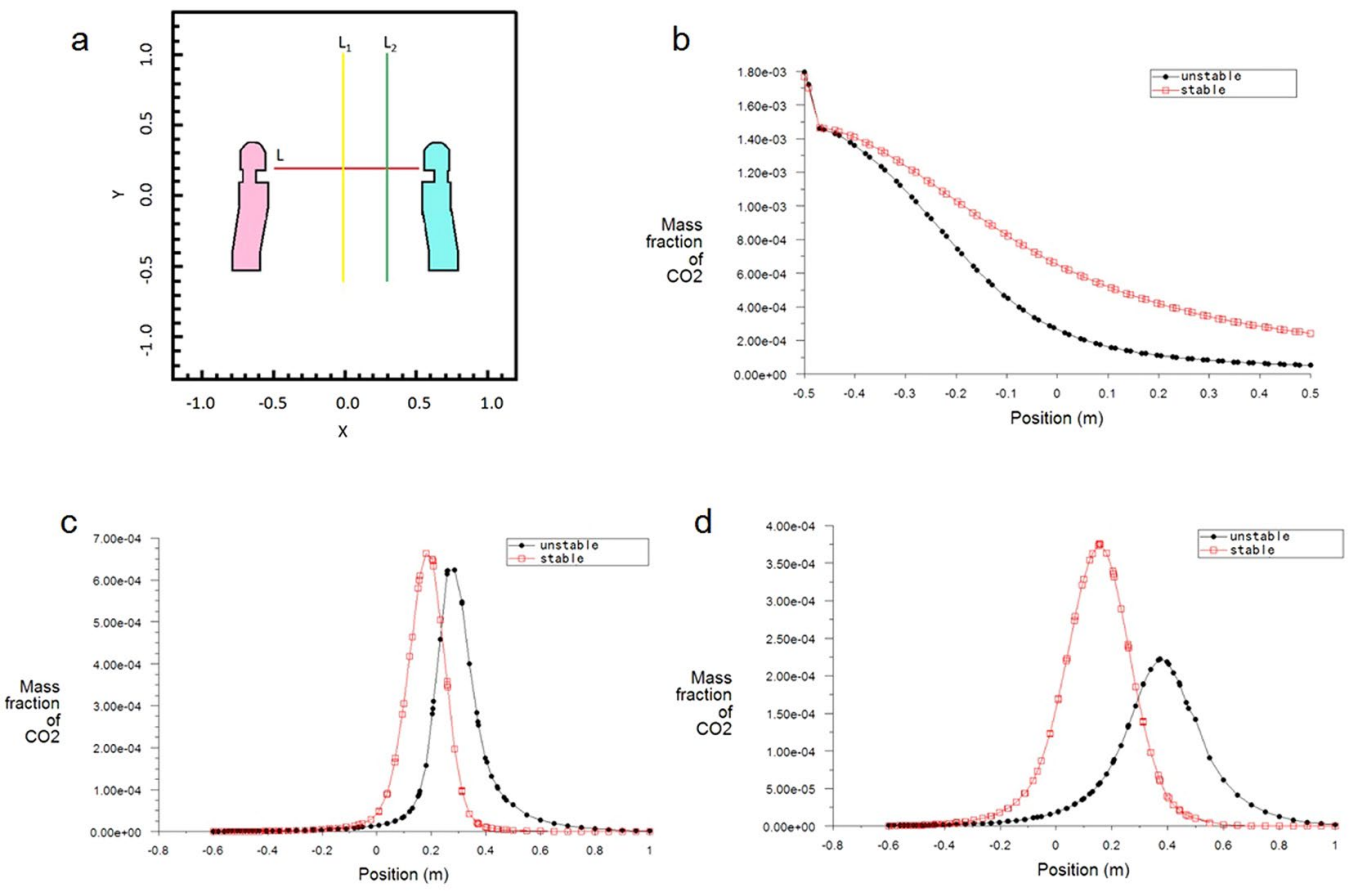
The characteristic of smoke concentration distribution in the dispersion direction under unstable case and stable case when t = 10 s. (**a**) Location map of manikins formed on the section of z = −0.45 m, (**b**) line graph along line L in **a**, showing that after a certain distance away from the mouth, the CO_2_ concentration in stable case is always higher than that in unstable case; (**c**) line graph along line L_1_ in **a**, showing that pollutants in unstable case continues to spread to the upper space, and a more obvious upward movement in Y direction; (**d**) line graph along line L_2_ in **a**, showing that in stable case, pollutants have a slight downward movement trend.

When t = 10 s, the peak concentration in stable case is higher than that in unstable case (Fig. 4), which indicates that compared with the couple or conjugating phenomenon in Fig. 1, turbulence diffusion, inertia transmission and accumulation phenomenon in Fig. 2, there is a kind of directional effect of transmission process under stable condition.

Thus, we find a kind of inertia conjugation effect that along the mainstream direction, the inertia transmission is strong, while in the other direction the inertia transmission process is weak. When the convective inertia transmission touches obstacle or human body, the inertia accumulation is more obvious because of rebounding and stagnating when air stability or inertia stability is stronger. When gravity does not exist, the simulation results of three conditions are the same. It means if there is no gravity, there is no inertia transmission process variation. Therefore, this study re-structures air stability process as inertia stability process, it shows that temperature background condition affects the interactive respiration process. And this work lays a theoretical foundation to healthy environment control, and may purify human beings behavior in public situation.

## Methods

There are three ways employed for temperature background effect via interactive respiration process. They are semi-analytical method, experiment analysis and CFD simulation, respectively. Amongst them, the experiment analysis used a size of 2.4 m × 1.8 m × 2.6 m room for performing air stability and a same size room is employed in CFD simulation.
